# Genetics of Familial Non-Medullary Thyroid Carcinoma (FNMTC)

**DOI:** 10.3390/cancers13092178

**Published:** 2021-04-30

**Authors:** Chiara Diquigiovanni, Elena Bonora

**Affiliations:** Unit of Medical Genetics, Department of Medical and Surgical Sciences, University of Bologna, 40138 Bologna, Italy; elena.bonora6@unibo.it

**Keywords:** non-medullary thyroid cancer, predisposing genes, next-generation sequencing

## Abstract

**Simple Summary:**

Non-medullary thyroid carcinoma (NMTC) originates from thyroid follicular epithelial cells and is considered familial when occurs in two or more first-degree relatives of the patient, in the absence of predisposing environmental factors. Familial NMTC (FNMTC) cases show a high genetic heterogeneity, thus impairing the identification of pivotal molecular changes. In the past years, linkage-based approaches identified several susceptibility loci and variants associated with NMTC risk, however only few genes have been identified. The advent of next-generation sequencing technologies has improved the discovery of new predisposing genes. In this review we report the most significant genes where variants predispose to FNMTC, with the perspective that the integration of these new molecular findings in the clinical data of patients might allow an early detection and tailored therapy of the disease, optimizing patient management.

**Abstract:**

Non-medullary thyroid carcinoma (NMTC) is the most frequent endocrine tumor and originates from the follicular epithelial cells of the thyroid. Familial NMTC (FNMTC) has been defined in pedigrees where two or more first-degree relatives of the patient present the disease in absence of other predisposing environmental factors. Compared to sporadic cases, FNMTCs are often multifocal, recurring more frequently and showing an early age at onset with a worse outcome. FNMTC cases show a high degree of genetic heterogeneity, thus impairing the identification of the underlying molecular causes. Over the last two decades, many efforts in identifying the susceptibility genes in large pedigrees were carried out using linkage-based approaches and genome-wide association studies, leading to the identification of susceptibility loci and variants associated with NMTC risk. The introduction of next-generation sequencing technologies has greatly contributed to the elucidation of FNMTC predisposition, leading to the identification of novel candidate variants, shortening the time and cost of gene tests. In this review we report the most significant genes identified for the FNMTC predisposition. Integrating these new molecular findings in the clinical data of patients is fundamental for an early detection and the development of tailored therapies, in order to optimize patient management.

## 1. Non-Medullary Thyroid Carcinoma (NMTC)

Non-medullary thyroid carcinoma (NMTC) is the most common form of thyroid cancer, accounting for more than 95% of cases [1]. NMTC originates from the follicular epithelial cells of the thyroid and represents the 1–5% of all tumor. However, in the last decades, it has become the most frequent endocrine neoplasia [2,3]. A family history of thyroid cancer, benign thyroid disease, obesity and smoking [3,4].

According to the latest WHO (World Health Organization) classification of thyroid tumors, and in concordance with the previous one released in 2004, thyroid neoplasia of follicular origin is classified in papillary versus follicular and benign versus malignant [5]. Follicular adenoma (FA) is defined as a non-invasive neoplasm showing follicular differentiation without papillary nuclear features [5,6].

The malignant forms can be classified on the basis of the histological features into papillary thyroid carcinoma (PTC), follicular thyroid carcinoma (FTC), poorly differentiated thyroid carcinoma (PDTC) and anaplastic thyroid carcinoma (ATC).

PTC is defined as a malignant epithelial tumor showing follicular differentiation with concrete nuclear characteristics [6]. PTC itself represents ~85% of all thyroid cancers [5,7,8]. FTC evolves from thyroid adenoma [9] and is defined as a follicular cell tumor without papillary nuclear features. FTC is less frequent than PTC, accounting for only 15% of all thyroid cancers [10] and is more frequent in women. FTC frequency is also increased in geographical areas poor in iodide [11]. FTC can present a morphological variant, defined the Hürtle cells carcinoma (HCC) or thyroid oncocytic carcinoma (TCO) [12]. However, in the new WHO classification, Hürtle cells tumors are considered as an independent group accounting for 2% of all thyroid malignancies [5,6,13].

Poorly differentiated thyroid cancer (PDTC) is a rare form of thyroid cancer, defined as a follicular cell tumor with limited evidence of follicular differentiation [5,14]. PDTC patients tend to present adverse clinical and histological features: older age, male predominance, advanced loco-regional disease, and distant metastases [15].

Anaplastic thyroid carcinoma (ATC) is the less frequent type of thyroid carcinoma, accounting for only 1–2% of all tumors. ATC is highly invasive and can derive from FTC, PTC or PDTC in which the follicular morphology of the cells is completely lost [16].

## 2. Familial Non-Medullary Thyroid Carcinoma (FNMTC)

NMTC often occurs in families, with a minimum of two affected relatives, although several families have been reported showing multigenerational cases [17,18]. Malchoff and Malchoff defined the familial form of NMTC (FNMTC) as the occurrence of the disease in two or more first-degree relatives of the patient, in the absence of predisposing environmental factors [19]. Among FNMTC cases, PTC is the most common histological sub-type (85–91%) reported, followed by PDTC (2–15%), FTC (6–9.7%), HCC (2%) and ATC (1.6%) [20].

FNMTC is increasingly recognized as a distinct clinical entity. Several researchers reported that familial NMTC cases are multifocal, bilateral, present lymph node metastasis, show a higher recurrent rate, an early age at onset and a decreased disease-free survival compared to sporadic forms [21,22,23,24,25]. Although still debated, it seems that FNMTC is more aggressive than the sporadic counterpart.

Familiarity is a significant risk factor for the development of thyroid cancer derived from follicular epithelial cells. In fact, the thyroid gland shows the highest familial relative risk compared to all other organs: as an example, the relative risk is 5–10 fold higher compared to breast and colon cancer [26].

FNMTC constitutes about 5–15% of all NMTC cases, including syndromic and non-syndromic forms [27]. Even if syndromic forms occur at low frequency, several genes responsible for different syndromes have been identified [28]. Syndromic forms include Cowden syndrome (due to causative variants in the gene *PTEN*), familial adenomatous polyposis (causative variants in *APC*) with its form Gardner syndrome, Carney complex (causative variants in *PRKARI*), Werner syndrome (causative variants in *WRN*)*,* DICER1 syndrome (causative variants in *DICER1*)*,* Ataxia-telangiectasia syndrome (causative variants in *ATM*) and Li-Fraumeni syndrome (causative variants in *TP53*) [29,30,31,32,33,34,35,36].

Nevertheless, the genetic alterations underlying the non-syndromic form are still largely unknown. There is still an undeniable lack of knowledge about the genetic causes of non-syndromic form, maybe due to the definition of FNMTC itself, since the inclusion of small pedigrees in the studies might lead to spurious associations [37]. In fact, the majority of FNMTC pedigrees are smalls in size and a potential bias in the diagnosis of FNMTC could be related with the presence, for example, of familial multinodular goiter (MNG) [38].

The identification of additional predisposing genes for familial NMTC is crucial to develop population prevention strategies and targeted management of the patient, as well as the possibility to identify individual with high risk to develop the disease.

## 3. Genetics of Familial NMTC (FNMTC)

### 3.1. Predisposing Loci Identified with Linkage Analysis and Candidate Gene Screening or Whole Exome Sequencing

A critical revision of the different families with individuals affected by NMTC suggested that FNMTC show an inheritance pattern compatible with an autosomal dominant character with variable penetrance or with a polygenic disorder likely associated with low-penetrant alleles [39] and a high degree of genetic heterogeneity. However, the mode of inheritance, monogenic or polygenic, is still debated [39].

In the last decades, linkage-based approaches have been applied on large pedigrees in order to identify susceptibility genes. Indeed, linkage intervals were identified for different genomic regions (on chromosomes 9p22.33, 14q32, 19p13.2, 2q21 and 1q21), and predisposing genes were identified in few of them [40,41].

#### 3.1.1. fPTC/PRN1 (1q21)

The fPTC/PRN1 locus (MIM 605642) on 1p13.2-1q21 was identified in an American kindred with multigenerational FNMTC and papillary renal neoplasia through linkage analysis obtaining a LOD score of 3.58 [42]. The authors sequenced the genes in the regions in the patients showing familial papillary renal cancer and thyroid cancer syndromes, but they did not find causative variants, and at this locus the causative gene is still uncharacterized.

#### 3.1.2. NMTC1 (2q21)

McKay et al. mapped a shared haplotype on chromosome 2q21 through a genome-wide scan in a large Tasmanian family with multiple individual affected by PTC [43]. Up to now, no candidate gene has been characterized at the 2q21 locus, named NMTC1 (MIM 606240).

#### 3.1.3. SRGAP1 (12p14)

The 12p14 locus was identified by genome-wide linkage analysis performed on 38 families with FNMTC. Even if the odd ratio identified was modest, direct Sanger sequencing in 21 families showing linkage to 12q14, led to the identification in 4 families of 4 germline missense variants in *SRGAP1*, co-segregating into the families with the PTC phenotype (p.Gln149His, p.Ala275Thr, p.Arg617Cys, and p.His875Arg) [44].

*SRGAP1* (Slit-Robo RhoGTPase activating protein 1) gene encodes for a GTPase activator, which interacts with the transmembrane receptor ROBO1 to inactivate CDC42. CDC42 is a small G-protein that regulates signaling pathways for different cellular functions such as cell morphology, migration, endocytosis and cell cycle progression, predicted to contribute to tumorigenesis [45]. To test the impact of *SRGAP1* variants, the authors co-transfected in a thyroid cell line the constructs encoding for wild-type and mutant *SRGAP1*, in combination with *ROBO1* and *CDC42* expression vectors. Functional studies revealed that in the presence of SRGAP1 missense variants p.Gln149His and p.Ala275Thr, CDC42-GAP activity was severely reduced and led to increased PTC invasiveness [44].

#### 3.1.4. MNG1 (14q32)

The 14q32 locus, MNG1 (MIM 138800), was first identified in a large Canadian family affected by multinodular goiter and PTC cases by microsatellite linkage analysis [46], but linkage to this locus was not replicated in additional 37 European and North America FNMTC families [46]. However, direct sequencing of the *DICER1* gene, mapping at this locus, in 53 members derived from five families (including the original one where linkage was first identified) with individuals affected by MNG and/or ovarian Sertoli-Leydig cell tumor (SLCT), led to the identification of germline causative variants [34]. Individuals affected by DICER1 syndrome (OMIM #601200) show a higher predisposition to thyroid cancer [47], Sertoli-Leydig cell tumors of the ovary (SLCT) [34] and pleuropulmonary blastomas [48].

*DICER1* encodes for a key member of the ribonuclease III (RNase III) family, involved in the generation of microRNAs (miRNAs). Micro RNASs are non-coding RNAs that have a role in the modulation of gene expression at the post-transcriptional level [34]. DICER1 contains two RNase III domains and a PAZ domain. The single-stranded RNA binding domain binds the 3′single-stranded overhang of the precursor miRNA. Interestingly, the length of the mature miRNA is determined by the distance of the bound miRNA precursor from the RNase III domains [49]. Khan et al. reported that individuals carrying *DICER1* causative variants showed a 16-fold higher risk to develop thyroid cancer in comparison to non-mutated individuals [47]. Moreover, Rutter et al. identified a germline heterozygous pathogenic causative variant in *DICER1* (p.Ser1814Leu) in six individuals of the same family presenting multiple cases of differentiated thyroid cancer and MNG [50].

#### 3.1.5. miR-886-3p and miR-20a

MicroRNAs (miRNAs) are short (20-22 nucleotides in lengths), highly conserved non-coding RNAs. By targeting mRNA, they can repress or enhance gene expression at the post-transcriptional level. Xiong et al. identified a differential expression of miR-886-3p and miR-20a by comparing miRNA profiles between familial and sporadic NMTC. MiR-886-3p and miR-20a were 3- and 4-fold overexpressed, respectively, in FNMTC [51]. Pathway analysis of genome-wide expression data from two different well-characterized thyroid cancer cell lines in which miR-886-3p was overexpressed revealed that miR-886-3p is involved in the regulation of DNA replication and focal adhesion [51]. Additional recent data have contributed to reinforce the role of miRNA dysregulated expression in the predisposition to thyroid cancer [52].

#### 3.1.6. SRRM2

Tomsic et al. performed a linkage analysis followed by Whole Exome Sequencing (WES) in a FNMTC family, and identified a heterozygous germline causative variant (p.Ser346Phe) in the *SRRM2* gene [53]. *SRRM2* encodes the serine/arginine repetitive matrix 2 protein, a splicing machinery subunit. The authors reported the presence of this variants also in sporadic NMTC and suggested that these predisposing variants may require additional environmental factors or other predisposing genetic factors for tumor development [53].

#### 3.1.7. MYO1F (19p13.2 Locus)

The 19p13.2 locus (MIM 603386), named TCO for thyroid carcinoma with cell oxyphilia, was mapped in a three-generation French family with an unusual form of FNMTC characterized by tumors with cell oxyphilia (also named Hürthle cells) [54]. Canzian et al. performed a parametric linkage analysis in this large French pedigree with six individuals affected by multinodular goiter/adenoma and three by carcinoma with cell oxyphilia. The tumor tissues were characterized by a high number of mitochondria that generated a large volume of granular eosinophilic cytoplasm, hence the definition of oxyphilia for this type of tumors. Considering a recombination ratio θ = 0 and a complete penetrance, the Authors mapped the susceptibility locus on chromosome 19p13.2 with a maximum LOD score of 2.97 [54]. Authors also excluded linkage to the known predisposing genes for thyroid tumors (*APC*, *PTEN*, *MNG1* and *TSHR*) and to the oncogenes mostly reported in sporadic NMTC (*RET*, *TRK*, and *MET*) [55].

**Figure 1:**
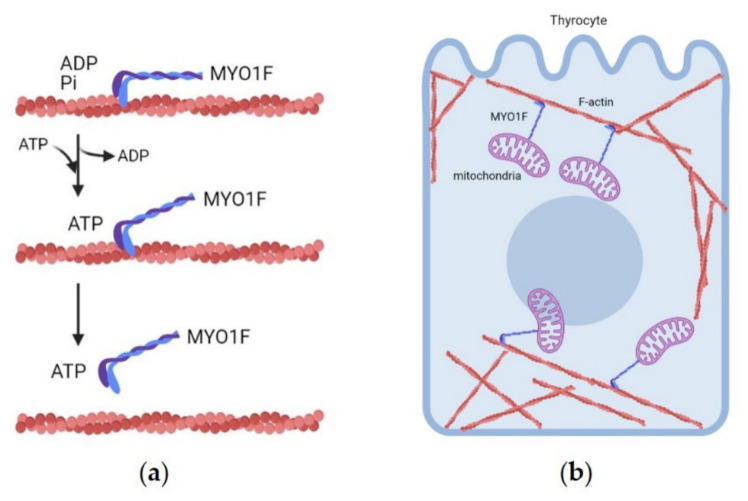
Molecular mechanism of MYO1F movements on actin filaments coordinating organelle transport. (**a**) MYO1F motor domain binds ATP and through the hydrolysis of adenosine triphosphate (ATP) produces the mechanical force along the actin cytoskeleton, (**b**) The figure represents a thyrocyte where MYO1F transports mitochondria along F-actin tracks, driving mitochondria in network. The causative variant p.Gly134Ser is predicted to alter the structure of the ATP binding domain in the molecular motor of MYO1F, blocking ATP hydrolysis and the movement of MYO1F towards actin filaments, with consequent alteration of the mitochondrial network. The figure was created with BioRender.com.

The causative variant segregating in the family was recently identified by whole exome sequencing (WES), i.e., a novel germline variant in the *MYO1F* gene inserting the missense change p.Gly134Ser [56]. In order to test the tumorigenic potential of mutant MYO1F, functional in vitro studies were performed in highly differentiated and functional rat thyroid cell lines stably expressing wild-type or mutant MYO1F [56]. The expression of p.Gly134Ser MYO1F increased the tumorigenic potential in terms of cell growth and invasion [56]. In vivo analysis in zebrafish confirmed that mutant p.Gly134Ser MYO1F, when overexpressed, could induce proliferation in whole vertebrate embryos. The mitochondrial structure and function was also studied in these cell models, considering that the tumor tissues of the patients carrying the *MYO1F* variant were characterized by mitochondrial hyperproliferation [54]. Live-cell analysis showed a fragmented mitochondrial network in mutant cells compared to controls, with an increase in the mitochondrial number, as in the original tumor tissues [56]. The variant p.Gly134Ser is predicted to alter the structure of the ATP binding domain in the molecular motor of MYO1F, blocking ATP hydrolysis and the movement of MYO1F towards actin filaments, which can lead to a disruption of the mitochondrial network (Figure 1). Moreover, mutant *MYO1F*- expressing cells showed an increase in reactive oxygen species (ROS) that activated the MAPK pathway [56]. Taken together, these data support the idea that *MYO1F* is the predisposing gene at the TCO locus.

#### 3.1.8. FTEN (8p23.1-p22) and 8q24

Two regions were identified on chromosome 8 through a genome-wide linkage analysis with high-density single nucleotide polymorphisms (SNPs) [57,58]. The first locus identified on chromosome 8 is the 8p23.1-p22 locus (FTEN). The FTEN locus was identified in a Portuguese pedigree, in which 11 individuals were affected by benign thyroid lesions and five by thyroid carcinomas. Cavaco et al. found a maximum parametric haplotype-based LOD (logarithm of the odds) score of 4.41 [58]. Linkage analysis with microsatellite markers confirmed linkage to 8q23.1-p22 and recombination events delimited the minimal region to a 7.46–Mb interval. Variant screening of 17 candidate genes in three patients with thyroid cancer from the original pedigree was performed, but no candidate variants were identified [58]. The authors sequenced the coding regions and splice sites of the candidate genes; however, they did not exclude the possibility of causative variants being present in regulatory elements or in intronic regions.

The second locus on chromosome 8, 8p24 was identified via linkage analysis with high density SNP arrays in different PTC families [57]. He et al. first studied a large three-generation family with PTC and melanoma, then extended the analysis to 26 additional PTC families. They identified a 320 Kb haplotype shared by the individuals with PTC, melanoma but not by unaffected individuals. The region comprises 2 known overlapping protein-coding genes, thyroglobulin (TG) and Src-like adaptor (SLA), but no putative causative variants have been identified by direct sequencing. Interestingly, the noncoding RNA gene AK023948 mapped in this region. There are evidences that the gene AK023948 might be a candidate gene for PTC susceptibility, since results from gene expression analysis, performed on PTC tumor tissues vs. controls, indicated a downregulation of AK023948 expression in most PTC tumors [57].

#### 3.1.9. MAP2K5

Ye et al. performed WES analysis followed by target sequencing of the candidate variants in a total of 77 FNMTC patients from 34 Chinese families affected by PTC [59]. Five patients harbored heterozygous germline causative variants in the *MAP2K5* gene. MAP2K5 is a dual specificity protein kinase that belongs to the MAP (mitogen-activated protein kinase) kinase family, which specifically interacts and activates MAPK7/ERK5 and was found to be the alternative pathway for MAP2K1/2–ERK1/2 [59]. Authors focused the analysis on two causative variants, c.G961A and c.T1100C (p.Ala321Thr and p.Met367Thr) both located in the MAP2K5 kinase domain [59]. They performed functional studies in the human thyroid carcinoma cell line B–CPAP stably expressing MAP2K5 p.Ala321Thr or MAP2K5 p.Met367Thr. They found an increased activation of the downstream protein ERK5 at the phosphorylation site Ser496 and Ser731/ Thr733 in the mutant cell lines compared to B–CPAP expressing the wild-type protein. ERK5 activation led to the active transcription of downstream targets *FOSB*, *MEF2*, *PPARG*, *CDK4*, and *CCND1* [59]. These data suggest that the alternative MAP2K5–ERK5 signaling pathway is likely a leading cause for FNMTC [59].

#### 3.1.10. NOP53

Orois et al. (2019) performed WES in four affected individuals of a FNMTC family and identified a variant in *NOP53*. Subsequently, they analyzed single nucleotide variants (SNVs), insertions and deletions in this gene in the affected members of 44 additional families with FNMTC [60]. They identified the variant c.91G > C, generating the missense change p.Asp31His, in *NOP53* gene (also known as *GLTSCR2*) present in heterozygous state and co-segregating with all affected members in three families. *NOP53* silencing showed inhibited cell proliferation and colony formation in vitro. Immunohistochemistry analysis on tumor samples from affected individuals, showed an increased NOP53 expression compared to normal adjacent thyroid tissue. Overexpression of mutant form of NOP53 increased cell proliferation and clonogenicity, supporting a growth promoting role [60]. NOP53 has a role in ribosomal biogenesis and functions as a nucleolar sensor that regulates the activation of p53/TP53 in response to ribosomal biogenesis perturbation, DNA damage and other stress conditions [60]. Evidences for a role of NOP53 in nuclear morphology, chromosomal stability and mitotic integrity support the hypothesis that it could be implicated in carcinogenesis events [61].

### 3.2. Predisposing Loci Identified via Genome-Wide Association Studies (GWAS)

In addition to the predisposing loci identified in FNMTC pedigrees, genome-wide case-control association studies (GWAS) have unveiled strong association signals for several SNPs in thyroid cancer predisposition.

#### 3.2.1. q22.33 Locus

##### FOXE1

Gudmundsson et al. carried out a landmark GWAS in the Icelandic population followed by a replication study in individuals of European origin, that led to the identification of two SNPs associated with increased risk of PTC and FTC in the general population [62]. The most significant variants identified as germline candidate risk factors were the SNPs rs944289, located on chromosome 14q13.3 near the gene *NKX2-1* and rs965513, located on chromosome 9q22.33 near the gene *FOXE1* [62].

Forkhead box E1 (*FOXE1*) gene encodes for a specific thyroid transcription factor (also termed thyroid transcription factor 2, TTF-2), important for thyroglobulin and thyroperoxidase gene expression and for the migration of thyroid cell precursors from the pharynx to the neck [63]. The *FOXE1* contains a polyalanine (polyAla) tract, characterized by the repetition of 11 to 22 alanine (Ala) residues, and the 14-Ala repeat is the most frequent allele in the general population.

In order to determine whether the sporadic NMTC-predisposing loci identified by Gudmundsson et al. (9q22.33 and 14q13.3) [62] could also be associated with increased risk of FNMTC, a high-throughput association study in a cohort of 238 families from Italy, France and Greece with at least two members affected with either PTC or FTC was performed. Single-SNP analysis was performed either on the nuclear families using the Family-based Association Test (FBAT) or on the whole pedigrees using the More Powerful Quasi-Likelihood Score (MQLS) method. The statistical analyses performed using FBAT, MQLS and logistic-normal model (LNM) confirmed the association between FNMTC and the two SNPs rs965513 and rs10759944 at 9p22.33 [64].

In support to the association between *FOXE1* and NMTC, direct sequencing of the *FOXE1* gene in 60 Portuguese FNMTC families and 80 sporadic NMTC cases led to the identification of 10 germline variants in the promoter and coding sequence of the gene [65,66]. The Authors generated wild-type and mutant constructs of *FOXE1* and performed functional studies on rat normal thyroid cells and human papillary thyroid carcinoma cell line, supporting a role of FOXE1 in tumorigenesis, since mutant FOXE1 promoted cell proliferation and migration [66]. These studies reinforced a role of FOXE1 variants in NMTC predisposition.

##### PTCS2-MYH9

A novel long intergenic non-coding RNA gene, termed papillary thyroid cancer susceptibility candidate 2 (*PTCSC2*) has been recently identified within the 9q22 locus and overlaps with the promoter region of the *FOXE1* gene. *PTCSC2* transcripts were downregulated in PTC tumors, in particular a lower expression of the unspliced *PTCSC2* and *FOXE1* transcripts was found in the affected individuals that were carriers of the risk allele [A] of the SNP rs965513, compared to carriers of the other allele [67].

Although the long noncoding RNA *PTCSC2* has been characterized in the locus along with the *FOXE1* gene, their interactions leading to carcinogenesis was unknown.

As cited above, individuals affected by PTC and carrier of the allele A of the SNP rs965513, had a lower expression of both *PTCSC2* and *FOXE1* [67]. The promoter region of *FOXE1* overlaps with the first exon and first intron of the isoform C of *PTCSC2*, but *PTCSC2* and *FOXE1* are transcribed in opposite directions [67]. Wang et al. investigated the PTC-promoting effect triggered by the associated variant at the rs965513 SNP and identified that myosin-9 (*MYH9*) was responsible for the regulation of the bidirectional promoter of *PTCSC2* and *FOXE1* [68]. MYH9 is a conventional non-muscle myosin, belonging to the II group of the myosin family. MYH9 regulates several functions, such as cell motility, cell-cell adhesion and maintenance of cell shape via actin filament-binding [69].

Wang et al. silenced *FOXE1* with siRNA in non-tumor human primary thyroid cells and performed a RNA deep sequencing in this model, identifying a perturbation of the p53 pathway that might be linked to tumor proliferation [68]. Therefore, they proposed that PTC predisposition, linked to the A allele of SNP rs965513, might be due to the interaction between the long non-coding RNA *PTCSC2*, its binding protein MYH9, and the gene *FOXE1*.

#### 3.2.2. NKX2-1 (14q13.3 Locus)

The second association signal identified by Gudmundsson et al. was for the risk allele T of the SNP rs944289, located on chromosome 14q13.3 near *NKX2-1* [62]. The *NKX2-1* gene encodes for a thyroid-specific transcription factor, binds to the thyroglobulin (*TG*) promoter and regulates the expression of thyroid-specific genes [70]. The SNP rs944289 maps in the promoter region of the long non-coding *PTCSC3*. *PTCSC3* expression in vitro was regulated by the binding of the activators C/EBP-α and C/EBP-β to *PTCSC3* promoter region and the risk allele T strongly decreased these interactions, reducing *PTCSC3* level of transcription, compared to the non-risk allele C [71]. Restoring *PTCSC3* expression in these cells inhibited cell growth and regulated the expression of target genes encoding for proteins involved in genome replication, recombination and repair, in cellular movement, cancer morphology and cell death [71].

Other studies identified the variant c.1016 C > T, inducing the missense change p. Ala339Val in NKX2-1, in two families with papillary thyroid carcinoma and multinodular goiter [72].

#### 3.2.3. DIRC3 (2q35 Locus)

Gudmundsson et al. in 2012 performed an additional GWAS in the Icelandic population, finding that SNPs in disrupted in renal carcinoma 3 (*DIRC3*) were associated with thyroid cancer. This study confirmed the association of 9q22.33 and 14q13.33 and led to the identification of a novel association to thyroid cancer for the SNP rs966423, located in the *DIRC3* gene mapping to the 2q35 locus [73]. The association at this locus was confirmed in another study published in 2017, where the authors found a strong association for the SNP rs11693806, mapping in the *DIRC3* gene [74]. Son et al. performed in 2017 the first GWAS in Asian population and confirmed the association of the *DIRC3* polymorphism rs11693806 with thyroid cancer [75].

### 3.3. Whole Genome Sequencing (WGS) Analysis

GWAS and linkage studies had been crucial in the identification of variants contributing to different human disorders, allowing the identification of both rare causative variants responsible for the Mendelian forms of disease, and common variants with weaker effects contributing to common disease susceptibility. Sequencing of the complete coding regions through next generation sequencing (NGS) approaches has uncovered causative variants in rare genetic disorders as well as predisposing variants in common diseases and cancer [76]. The recent advances in NGS have led the introduction of whole genome sequence (WGS) analysis in cancer genetics. WGS studies in FNMTC families are therefore a strategy to identity new predisposing genes. However, the application of WGS leads to the detection of a large amount of genetic data, requiring specific pipeline to filter and select variants. Recently, Kumar et al. published the Familial Cancer Variant Prioritization Pipeline (FCVPPv2), a standardized protocol to interpret WGS data and to identify pathways that might be dysregulated in the FNMTC families, facilitating the prioritization of predisposing germline variants with high/moderate-penetrance even in relatively small families [77].

#### 3.3.1. CHEK2

Wójcicka et al. demonstrated a significant association for the SNPs rs17879961 in *CHEK2* (encoding for checkpoint kinase 2) and rs16941 in *BRCA1* with PTC susceptibility. Siołek et al. (2015) detected a statistically significant correlation between individuals harboring the c.1100delC, c.444+1G > A, c.del5395 *CHEK2* variants and increased risk of PTC. To further characterize the association between these variants and the predisposition to thyroid cancer, they genotyped *CHEK2* in 468 unselected patients with PTC, detecting causative variants in 73 of the 468 (15.6%) unselected PTC patients [78].

Through WGS analysis, we recently identified two potentially disease-causing variants in *CHEK2* and EWS RNA Binding Protein 1 (*EWSR1*) in a FNMTC family with patients showing PTC, micro-PTC, and insular carcinoma [79]. The variant identified in *CHEK2* has been already reported in breast and prostate cancer.

*CHEK2* gene encodes for a cell cycle checkpoint regulator tumor suppressor involved in DNA damage-induced DNA repair, cell cycle arrest, and apoptosis. In mammalian cells, when a damage in the genomic DNA occurs, the ATM kinase directly activates CHEK2. Activated CHEK2 in turn activates BRCA1, leading to DNA repair or apoptosis. Alterations of this axis lead to tumorigenic changes [80]. CHEK2 and EWSR1 both converge in the DNA-damage repair pathway, therefore we hypothesized that in this family they might act in combination for the development of thyroid cancer [79].

#### 3.3.2. Telomere Abnormalities

In recent years, the role of telomeres in the regulation of cell life span has been clarified, and it has strongly emerged as related to NMTC pathogenesis.

Being linear, the ends of chromosomes might be considered by the DNA repair machinery as DNA double-strand breaks (DSBs). In order to prevent the activation of sentinel proteins involved in DSB repair mechanisms, the ends of linear chromosomes in eukaryotes contain caps, termed telomeres [81]. In mammalians, telomeres are composed by the TTAGGG repetitive sequence with a folded final telomeric loop (t-loop) composed by short single-stranded 3′ overhang [82,83,84].

Telomerase is insufficiently expressed in mammalian somatic cells, and for each cell division 50 to 200 nucleotides of telomeric repeats are lost [85]. During DNA replication the RNA primer does not bind to the very beginning of the chromosome, and the ends of the chromosome are being processed to form the cap. Due to these two phenomena the telomere becomes shorter at each cell division [85]. When the telomere becomes too short, the loop is disrupted and dysfunctional telomeres mimic a DNA damage, activating the DNA damage response (DDR) cascade [84], to prevent mutational events.

A complex system of accessory proteins cooperates to maintain the equilibrium. Among them, the shelterin protein complex, composed by TRF1, TRF2, RAP1, TIN2, TPP1, and POT1, that is located on t-loop, plays a pivotal role in the protection of chromosome ends from DNA damage repair mechanisms [86].

Telomeric repeat binding factor 1 (TRF1), telomeric repeat binding factor 2 (TRF2), and protection of telomeres 1 (POT1) directly recognize and bind TTAGGG repeats. Together with adrenocortical dysplasia protein homolog (ACD, also known as TPP1), TERF1-interacting nuclear factor 2 (TIN2), and telomeric repeat binding factor 2 interacting protein (TERF2IP) form a protein complex. Although TIN2 does not directly bind to telomeric DNA, it is a critical part of shelterin through its interactions with TERF1, TERF2, and ACD, allowing the complex to differentiate telomeres from sites of DNA damage.

In recent years, the concept that telomere length is connected with susceptibility to PC has been reinforced [87], in particular a shorter relative telomere length (RTL) has been observed in patients with FNMTC [88,89,90], as well as the description of genetic variants in the genes of shelterin complex as reported below.

A loss-of-function causative variant in *TINF2* (p.Trp198fs) was identified in a family with melanoma and thyroid cancer predisposition. This disruptive causative variant in *TINF2*, that prevented the binding and activation of TERF2, induced the formation of longer telomeres [91]. The authors screened the coding sequence of the six shelterin genes in 24 families and identified two missense variants in *TINF2* and one in *ACD*, predicted damaging in silico by SIFT (https://sift.bii.a-star.edu.sg/ accessed date: 29 April 2021), PolyPhen-2 (http://genetics.bwh.harvard.edu/pph2/ accessed date: 29 April 2021) and VEP (https://www.ensembl.org/Tools/VEP accessed date: 29 April 2021), although no additional functional studies were performed [91].

Another study identified the *POT1* missense variant p. Val29Leu in one FNMTC pedigree, variant predicted to interfere with the interaction between POT1 and ACD [92]. A reduction in telomere-bound POT1 levels and increased telomere length were observed in the mutant cells in comparison to wild-type cells [92].

A causative variant in *POT1* have also been reported in a pedigree with predisposition to several tumors [93]. Recently Richard et al. highlighted a connection between an intronic regulatory variant in *POT1* and the risk to develop thyroid carcinoma, suggesting the idea that this regulatory *POT1* variant might be responsible for longer telomeres [94].

Although alterations in the shelterin complex genes seem to be rare events, the reported data suggest that when they are altered, these proteins can cause a telomere disruption and may play a role in predisposition to FNMTC.

**Figure 2:**
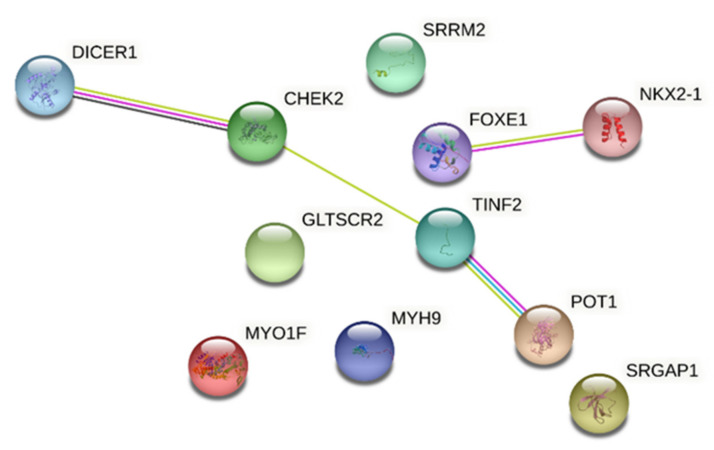
Network analysis of FNMTC-predisposing gene products. The figure shows the proTable 1. *SRGAP1, SRRM2, MYO1F, FOXE1, MYH9, NKX2-1, CHEK2, NOP53 (GLTSCR2), TINF2* and *POT1.*

Taken together, the genes involved in FNMTC predisposition encode for a wide variety of proteins and cell functions, indicating that different hits may converge in this type of cancer. We performed a protein network analysis with STRING (https://string-db.org/ accessed date: 29 April 2021) [95] of the principal players involved in NMTC predisposition. We observed that these proteins had more interactions among them than expected for a random group of proteins with similar size in the human genome (PPI (Protein-Protein Interaction) enrichment *p*-value = 0.043, Figure 2).

This hit indicates that the proteins are at least partially biologically connected, as a group. In a total of 11 edges, four nodes were found. The Homeobox protein Nkx2.1 (*NKX2-1*) is associated with the forkhead box protein E1 (*FOXE1*), both thyroid-specific transcription factors [96]. FOXE1, containing the DNA-binding “forkhead” domain, is able to bind and unroll the chromatin structure allowing the access to others DNA transcription factors [97]. NKX2-1 is a thyroid-specific transcription factor through the binding to the thyroglobulin promoter and the regulation of the expression of genes involved in thyroid morphogenesis [98].

It is interesting to note the clustering of DICER1, CHEK2, TINF2 and POT1. Performing a functional enrichment based on gene ontology with Panther [99,100], we found a hit for the telomeric function, with a False Discovery Rate (FDR) = 0.0016 for telomere assembly in Biological Processes, FDR = 0.0247 for telomeric DNA binding in Molecular Function, FDR = 0.0011 for shelterin complex in Cellular Component.

## 4. Discussion

We provide a thorough review of the literature for the most significant candidate genes implicated in the genetic predisposition to NMTC either in families or as constitutive predisposing genetic factors (Table 1).

At the histopathological level, thyroid tumors of epithelial origin have been distinguished in well-differentiated thyroid carcinomas which include papillary thyroid carcinomas, follicular thyroid carcinomas, and Hürthle cell thyroid carcinomas, and less differentiated neoplasia, such as poorly differentiated thyroid carcinomas and anaplastic thyroid carcinomas. However, the molecular genomic alterations found in the different histological subtypes, but also within them, can be variable, with a consequent clinical impact in terms of prognosis and survival. Indeed, the presence of secondary mutations can define a subset of aggressive tumors, often resistant to standard treatments [101,102]. In this framework, the evidence of familiarity and the identification of the predisposing factors that may promote the occurrence of the somatic mutations would greatly improve population surveillance and identification of at-risk patients.

However, several advances are still required to fully elucidate the etiology of FNMTC, considering that this type of cancer is influenced by genetic and environmental factors. FNMTC has an autosomal dominant mode of inheritance with incomplete penetrance and variable expression [103]. Moreover, FNMTC cases are characterized by high genetic heterogeneity, complicating the identification of pivotal molecular constitutive genetic risk factors.

The emerging new technologies of DNA sequencing have helped the expansion of the knowledge of the genetic predisposition of FNMTC, leading to the identification of a whole new spectrum of variants. However, the high heterogeneity for this type of cancer has hampered a robust replication of these data and a major predisposing gene has not been identified so far.

In particular, it is critical to identify aggressive cases with poor prognoses, but at the same time over-diagnosis or overtreatment of low-grade disease or benign nodules should be avoided [40]. These opposite needs can be fulfilled only when there is a deep understanding of predictive germline variants and their underlying pathways. Nevertheless, it is evident from literature that many ultra-rare disease-causing loci have yet to be discovered and there is an intrinsic problem in focusing the prioritization on one damaging variant rather than on another in pedigrees showing different variants in pathogenic potential in silico. However, the discovery of additional genes predisposing to familial NMTC is warranted to improve prevention strategies and targeted management of the patients. The recent introduction of WGS analysis allows the concurrent evaluation of single nucleotide/small insertion-deletions variants as well as structural variants in the entire genome, with reduced time and costs, covering the whole spectrum of constitutive genomic alterations that may predispose to diseases. Integrating these analyses with the clinical data of patients and their families will be fundamental for an early diagnosis and an optimized patient management.

## Figures and Tables

**Table 1:** Susceptibility loci and candidate genes linked to NMTC.

Chromosomal Position	Locus Name	Candidate Gene	Reference	Technique
1q21	fPTC1/PRN1	-	[42]	**Linkage Analysis/WES**
2q21	NMTC1	-	[43]	
12p14	-	*SRGAP1*	[44]	
14q32	MNG1	*DICER1*	[34,46]	
	-	*miR-886-3p and miR-20a*	[51]	
16p13.3	-	*SRRM2*	[53]	
19p13.2	TCO	*MYO1F*	[54,56]	
8q24	-	AK023948	[57]	
8p23.1-p22	FTEN	*-*	[58]	
15q23	-	*MAP2K5*	[59]	
19q13.33	-	*NOP53*	[60]	
9q22.33	-	*FOXE1*	[62]	**GWAS**
9q22.33	-	PTCS2	[67]	
9q22.33	-	*MYH9*	[68]	
14q13.3	-	*NKX2-1*	[62,72]	
2q35	-	*DIRC3*	[73,74,75]	
22q12.1	-	*CHEK2*	[79]	**WGS**
14q12	-	*TINF2*	[91]	
7q31.33	-	*POT1*	[92]	
